# Economics of Musculoskeletal Ultrasound

**DOI:** 10.1007/s40134-016-0169-5

**Published:** 2016-06-20

**Authors:** Nathalie J. Bureau, Daniela Ziegler

**Affiliations:** Department of Radiology, Centre hospitalier de l’Université de Montréal, 1058 St-Denis street, Montreal, QC H2X 3J4 Canada; Research center, Centre Hospitalier de l’Université de Montréal, Montreal, QC Canada; Library, Centre Hospitalier de l’Université de Montréal, Montreal, QC Canada

**Keywords:** Economics, Musculoskeletal system, Imaging, Ultrasonography, Magnetic resonance imaging, Healthcare costs

## Abstract

**Purpose of review:**

Healthcare costs have exploded in the past 30 years and they are a major concern for governments worldwide. Care management of musculoskeletal disorders and advanced imaging account for a large part of this socioeconomic burden.

**Recent findings:**

Musculoskeletal ultrasound is now performed primarily by nonradiologists. Both musculoskeletal ultrasound and MRI total utilization rates continue to increase. Despite the existence of evidence-based diagnostic recommendations and the potential cost-savings of using musculoskeletal ultrasound instead of MRI in certain clinical situations, ensuring appropriate use of imaging among health professionals remains difficult for various reasons.

**Summary:**

In the context of healthcare budgets restraints, use of imaging must be shown scientifically, to improve patient outcomes and be cost-effective. Current evidence recommends musculoskeletal ultrasound as the primary imaging modality in the investigation of rotator cuff disease. Policies aiming at ensuring the application of imaging guidelines among physicians are needed.

## Introduction

In all countries, there is pressure on budgets dedicated to healthcare and social resources that has been steadily increasing in the past 30 years, notably because of the aging of the population [1]. Musculoskeletal (MSK) disorders contribute a large part of this socioeconomic expenditure; therefore, many are concerned with the burden of MSK disorders. To improve MSK health of populations globally, studies have suggested that strategies should be directed to the prevention of recognized risk factors including: obesity, poor physical fitness, smoking, excessive alcohol consumption, a diet lacking in essential nutrients, calcium and vitamin D, and work-related, road traffic, and sporting injuries [2, 3]. In parallel, early diagnosis and appropriate care aiming at reducing pain and minimizing the sequelae of MSK diseases should be achieved in the most cost-effective way. This requires that patients have timely access to healthcare professionals with adequate competency [4], to outcome-impacting, cost-effective diagnostic algorithms and to up-to-date, evidence-based care management.

**Fig. 1:**
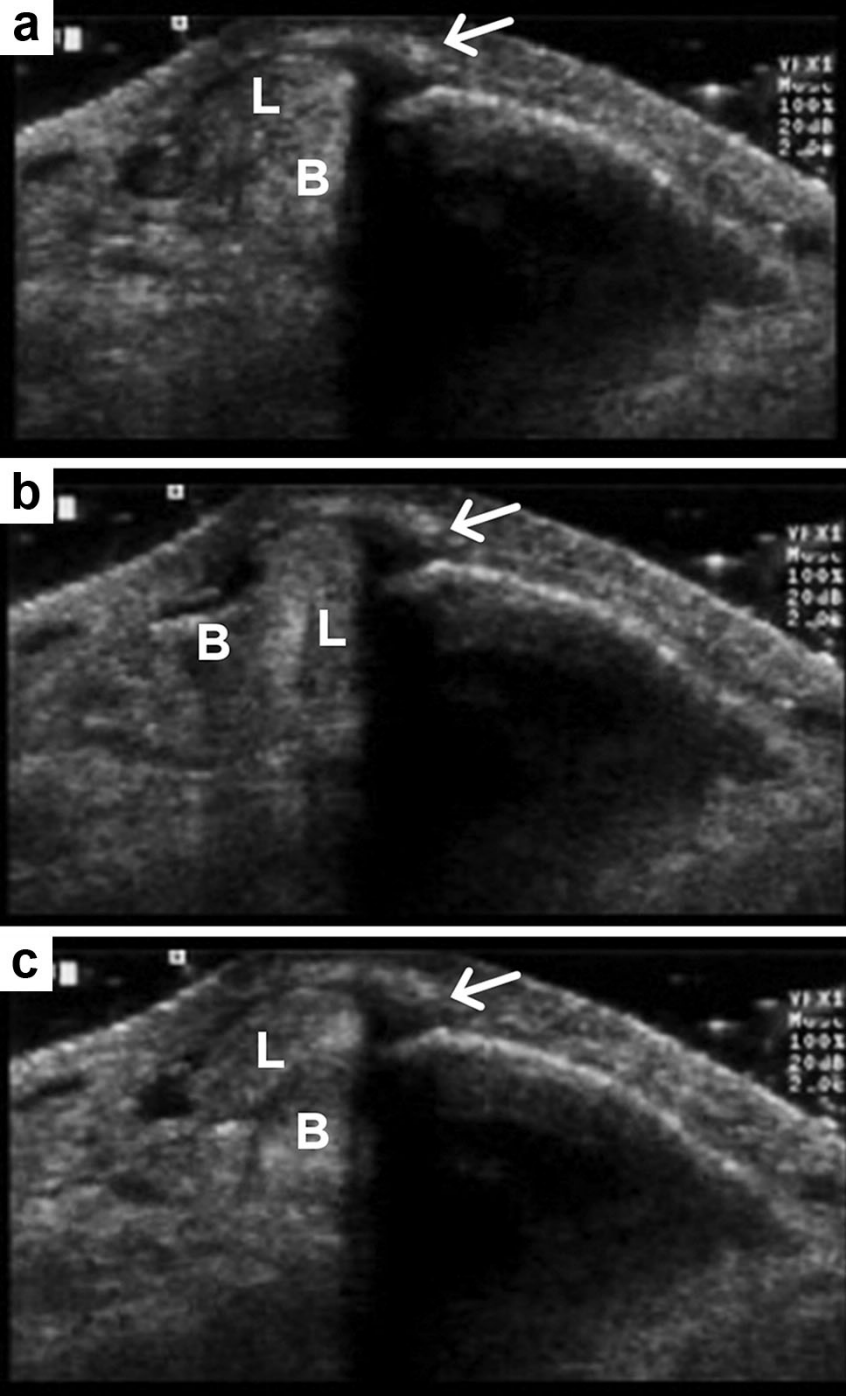
Retromalleolar intrasheath peroneal tendon subluxation in a young downhill skier occult at MRI and demonstrated at dynamic ultrasound examination. **a** Transverse ultrasound scan at the level of the lateral malleolus with the ankle at rest shows the peroneus longus (L) and peroneus brevis, (B) tendons in anatomical position. The *arrow points* at a mildly thickened superior retinaculum. **b** During active ankle eversion, the tendons abruptly interchange position within the retromalleolar groove. Note the increased laxity of the superior retinaculum (*arrow*). **c** As the ankle is brought into neutral position, the tendons suddenly switched back to their anatomical position. (*arrow* = superior retinaculum)

Another contributing factor in the escalating cost of healthcare is the use of advanced imaging [5]. The diagnostic imaging armamentarium available to physicians for the investigation of MSK disorders comprises primarily: radiographs, computed tomography (CT), ultrasound, magnetic resonance imaging (MRI), and nuclear medicine studies. Because of its unsurpassed spatial resolution and dynamic imaging capabilities, applications for MSK ultrasound have broadened significantly over the years making ultrasound an effective, less-costly alternative to MRI in many clinical situations, and the imaging test of choice in others (Fig. 1). While advocating utilization of MSK ultrasound over other more expensive imaging modalities such as MRI could be viewed as an effective way to reduce healthcare costs, many issues related to accuracy, observer variability, training and certification, and use by nonradiologists have to be considered along with pure economics [6, 7].

This review summarizes the recent literature related to the economics of MSK imaging, with an emphasis on ultrasound, and the impact on diagnosis, care management, and improved health of MSK disorders.

## Literature Search for Identification of Relevant Papers

The search strategy used combined words and expressions for these three conceptual groups: (1) economics, (2) musculoskeletal system, and (3) imaging. We used words and expressions from controlled vocabulary (MESH, EMTREE, etc.) and free text searching in order to identify studies in CINAHL (from 1937 onwards), EMB Review (from 1991 onwards), EMBASE (from 1974 onwards), MEDLINE (from 1946 onwards), PubMed, and the grey literature (MedNar Database, TRIP Database, Google Scholar, CADTH, National Guideline Clearinghouse, National Institute for Health and Care Excellence, National Institute for Health Research, OpenGrey and WordCat.). We provided a detailed report of the search strategy translation used in each database. The results from multiple searches were merged using the reference management software EndNote (version X7.4) and duplicate records were removed. The search yielded 3031 results. The first author scanned the titles of all retrieved publications and when necessary, also read the abstracts. Forty-seven papers published over the past 20 years were deemed relevant to the topic of this review article and were read in preparation on the present paper. In addition to the electronic search strategy, searching the reference lists of the retained papers identified other relevant publications.

## Burden of MSK Disorders

Musculoskeletal disorders are extremely common across all ages in industrialized and developing countries. These conditions are among the leading causes of pain and physical disability. They represent a diverse group of pathologies including inflammatory diseases such as rheumatoid arthritis (RA) and other arthriitdes; degenerative and microtraumatic diseases such as osteoarthritis and tendon disorders; conditions related to traumatic, sports, and occupational injuries; and other common disorders such as back pain. Some of these conditions are recurrent; others are acute and of short duration but many are chronic with increasing severity over time. Furthermore, the prevalence of many of these disorders increases with age.

It is difficult to capture the real impact of MSK disorders on individual well-being and societal healthcare costs and resources as statistics, which have been traditionally used to measure the societal burden of disease, are biased towards diseases with high mortality and underestimate the onus of MSK conditions [8]. MSK conditions are associated with high morbidity but low mortality and are extremely prevalent in the working population [9]. At the individual level, MSK disorders cause pain and physical impairment that restrict activities of daily living. Work loss and direct health expenditures related to care management are the most significant socio-economical burdens resulting from MSK diseases [10].

## Evolution of MSK Ultrasound

Since the first description of the technique of ultrasound examination of the normal rotator cuff by Middleton et al. [11] in 1984, there have been a number of technical advances that have influenced the practice of MSK ultrasound. Higher-frequency linear transducers, compound imaging, tissue harmonic imaging, and extended-field-of-view, to name a few, have improved ultrasound imaging of joints and soft tissues, especially as it pertains to superficial structures such as tendons, ligaments, and nerves. MRI offers a comprehensive evaluation of bone, cartilage, and intra-articular structures and certainly remains the imaging reference standard for a variety of MSK disorders. On the other hand, from a radiologist’s point of view, MSK ultrasound offers specific advantages over MRI, as described by Nazarian [12] in his article, which defines the top ten areas where MSK ultrasound demarcates itself. For instance, contrary to MRI, there are no contraindications, such as cardiac pacemakers and other body implants, to MSK ultrasound. Because of its higher spatial resolution, MSK ultrasound can resolve finer anatomic details than MRI. MSK ultrasound is also unique in its capacity to perform dynamic imaging, to correlate findings with the patient’s symptomatology and in guiding procedures allowing achieving diagnosis and treatment during one clinical session. Versatility in probe positioning allows examining long anatomic segments, which is proving very useful for the investigation of peripheral nerves [13]. MSK ultrasound coupled with Doppler ultrasound and more recently, with elastography can provide pertinent physiologic information. Moreover, in certain clinical situations, such as the evaluation of rotator cuff tears, ultrasound provides diagnostic accuracy comparable to MRI and MR arthrography as reaffirmed in a recent meta-analysis [14]. Hence, MSK ultrasound should be regarded as an imaging modality that can be, depending on the patient’s clinical presentation, either complementary to MRI, or an alternative to MRI, or even more appropriate than MRI.

More recently, the advent of compact, portable, and more affordable ultrasound machines has democratized the utilization of MSK ultrasound beyond the traditional confines of imaging departments. Rapidly, the use of MSK ultrasound as a diagnostic and intervention-guiding technique has gained acceptance in the fields of rheumatology, orthopedic surgery, physiatry, and podiatry [15, 16]. The perspective of nonradiologists performing MSK ultrasound is that it is complementary to history and physical examination and can enhance the ability to provide more effective and efficient patient care [17]. Furthermore, it increases the technical accuracy of interventional procedures [18], and according to some authors, it may decrease the use of MRI examinations [19].

## Training and Certification in MSK Ultrasound

When incorporating MSK ultrasound into their practice, to ensure high standards of patient care, radiologists and nonradiologists alike must address several issues [20, 21]. First the training in MSK ultrasound is challenging. Should teaching of MSK ultrasound be incorporated at the residency level and can it be? In our experience, at the present time, there is limited exposure to MSK ultrasound during the residency in radiology and MSK ultrasound is more likely to be taught during postgraduate fellowship training. In the case of practicing physicians, courses are offered by several organizations and self-teaching of anatomy, pathology, and scanning protocols can be done through web-based curriculum and scanning guides as offered for instance by the American Institute of Ultrasound in Medicine (AIUM) (http://www.aium.org), the European Society of Musculoskeletal Radiology (ESSR) (http://www.essr.org), and the European League Against Rheumatism (EULAR) (http://www.eular.org). Besides the important commitment that this self-training requires, according to a Canadian study, nonradiologists may also face other challenges of time restraints related to clinical workflow, costs associated with equipment acquisition, and the ability to receive remuneration for MSK ultrasound examinations [22].

MSK ultrasound should be performed using standard scanning techniques and protocols with image documentation and a written report for quality assurance. The question of certification and accreditation should also be addressed, as training alone does not guarantee competency. In that regard, the American College of Rheumatology has been proactive in defining the role of MSK ultrasound in the rheumatologist’s scope of practice and establishing the pathways of certification [15], whereas the AIUM is now accrediting practices for MSK ultrasound.

## MSK Ultrasound Utilization

In an effort to appraise the changes in practice generated by the increase in the number and diversity of MSK ultrasound providers, Sharpe et al. [23] looked at the trends of MSK ultrasound utilization in the United States of America (USA) over the first decade of this century. The authors reported a +316 % increase in the total number of diagnostic MSK ultrasound examinations paid under Medicare Part B, between 2000 (56,254 studies) and 2009 (233,964 studies) [23]. Of the total MSK ultrasound utilization, radiologists performed 40,877 (72.7 %) ultrasound exams in 2000 and 91,022 (38.9 %) in 2009, accounting for a +123 % increase. During that same time period, podiatrists experienced the most dramatic increase in MSK ultrasound volume (+1847 %) than any other type of care provider, including radiologists, rheumatologists, general practitioners, and all other providers combined. Of the total MSK ultrasound volume, podiatrists performed 3920 (7.0 %) procedures in 2000 and 76,332 (32.6 %) procedures in 2009, in second place to radiologists. This article also informs us that between 2000 and 2009, the increase in MSK ultrasound examination volume varied by practice setting, expanding mostly in private offices (+717 %) followed by hospital outpatient facilities (+102 %). Podiatrists were responsible for 51.5 % of this growth in private office MSK ultrasound utilization, whereas rheumatologists claimed 16.1 %, radiologists claimed 9.2 % and all other providers combined claimed the remaining 23.2 % of this growth, respectively. Another interesting element of this economic analysis by Sharpe et al. [23] is the comparison between MSK ultrasound and MSK MR examination utilization during that time period. According to their analysis, the total utilization rate of MSK ultrasound increased from 171 per 100,000 in 2000, to 669 per 100,000 in 2009 (+291 %), while the corresponding figures for the gross utilization rate of MSK MR studies were 1421 per 100,000 in 2000 and 3668 per 100,000 in 2009 (+158 %). The authors arrive at the conclusion that the parallel significant increase in both MSK ultrasound and MSK MR examinations between 2000 and 2009 most likely reflects a net increase in overall imaging studies and not a trend for substitution of MSK ultrasound for MSK MR examinations.

Another question that arises concerning MSK ultrasound utilization is: who should perform imaging? Most imaging done by nonradiologists is self-referred, whereas radiologists in their capacity as consultants are generally not in a position to self-refer and may advise appropriate, cost-effective imaging. A study showed that Medicare reimbursement to nonradiologists for noninvasive diagnostic imaging grew more rapidly between 1998 and 2006 than for radiologists and that in 2008, nonradiologists received 4 % more Medicare payments than radiologists for these studies [24]. Kennedy et al. [25] showed that after the application of the Deficit Reduction Act, which reduced Medicare payment for selected in-office procedures beginning in 2007 in an attempt to contain the constant growth of imaging healthcare costs, the growth rate of in-office noninvasive MSK imaging performed by nonradiologists continued to increase more rapidly that that performed by radiologists.

## Appropriate Use of Imaging

This demonstration of the tremendous increase in MSK ultrasound utilization over a period of 10 years raises the interrogation of appropriateness of utilization. In other words, are these imaging studies justified because they improve health outcomes of patients? Is the performance of these imaging studies driven by a concern for cost-effective care management? Do evidence-based diagnostic guidelines exist and do MSK ultrasound providers follow them?

In 2013, the Society of Radiologists in Ultrasound published a consensus conference statement on the recommendations for imaging of patients suspected of having rotator cuff disease [26]. Based on the available literature reporting an almost equivalent accuracy of MSK ultrasound and MRI for full and partial-thickness rotator cuff tears and considering the lower cost of ultrasound and its greater patient acceptance, the panel of experts agreed that MSK ultrasound should be the primary examination in the case of suspected rotator cuff disease in the native shoulder [26]. Despite the existence of these guidelines and the promotion of the value of MSK ultrasound [27], MRI is still widely performed in lieu of MSK ultrasound in that clinical setting [28]. This is well demonstrated in a study by Yeranosian et al. [29] who examined the cost expenditures associated with the preoperative diagnostic evaluation and conservative treatment of patients ultimately undergoing primary rotator cuff repair in the USA between the years 2004 and 2009. The authors looked at the charges billed to insurance providers for outpatient physician visits, diagnostic imaging studies, injections, physical therapy, laboratory, and other preoperative studies in the 90-day period preceding surgery. The largest expenditure category was diagnostic imaging studies, including MR studies, radiographs, arthrograms, and other unrelated imaging studies, which accounted for $104,510,646 representing 65 % of the total charges. MR examinations accounted for $91,434,079 (88 %) of all imaging studies and represented the single most expansive preoperative expenditure. Evidently, MSK ultrasound was underutilized in that cohort of patients, despite the existence of diagnostic algorithm recommendations and the potential cost-savings of using MSK ultrasound instead of MRI. Regarding cost-effectiveness, Parker et al. [30] investigated the use and costs of MSK imaging in the United States of America (USA) Medicare population between 1996 and 2005 and found that during that time period, MSK MRI increased by 353.5 % while MSK ultrasound increased by 157.1 %. The authors projected this trend from 2006 to 2020 and estimated cost-savings that could be anticipated by substituting MSK ultrasound for MSK MRI, when appropriate. According to their projection model, cost-savings to Medicare during that 15-year period could exceed $6.9 billion [30].

These studies illustrate the difficulty in ensuring appropriate use of imaging among health professionals [31, 32]. Another example is provided by the study of Griffith et al. [33•] who looked at the appropriateness of the use of cervical spinal CT in the emergency department, in patients who had sustained blunt trauma. In the first phase of their study, the authors retrospectively looked at the indications for performing the CT examinations based on recognized appropriateness criteria. Of 1524 studies with negative findings, 364 (23.9 %) did not meet the guidelines and could have been avoided. The authors identify possible causes of inappropriate use of imaging in that clinical setting including: lack of knowledge or awareness of guidelines, failure to recall the guidelines, lack of trust in the guidelines, complexity of the guidelines making them difficult to adhere to, or clinical judgment superseding the guidelines [33•]. In 2009, experts participating in the American Board of Radiology Foundation summit, on the causes and effects of the overutilization of imaging, identified other factors that may influence inappropriate use of medical imaging including: the fee-for service payment system; financially motivated self-referral practices; defensive medicine; the lack of comparative effectiveness research studies establishing evidence of the value of imaging modalities; patient expectations; and duplication of imaging studies [34].

## Economic Evaluation Studies

In 2009, a large American insurance company declared nonoperative spinal and MSK ultrasound experimental with the consequence of denying reimbursement to the radiologists and nonradiologists who perform MSK ultrasound [35]. This policy aiming at controlling overutilization of MSK ultrasound and its costs was overturned after individuals and several health care organizations made representations. Similarly, more recently in France, reimbursement for MSK MRI interpretation was cut down in an effort to decrease imaging costs [36]. These situations indicate how healthcare costs are under scrutiny by policy makers and other actors looking to control health care expenditures and the importance for imaging providers to be knowledgeable in imaging and healthcare service costs [37] and to offer clinical effectiveness and quality imaging [35].

Economic evaluation studies assess both costs and outcomes of health care interventions. These evaluations may take the form of cost-minimization, cost-effectiveness, cost-utility and cost-benefit analyses. In 2012, Ifedayo et al. [38] published a systematic review on economic evaluations in shoulder pathologies and found 32 articles that were published on this subject between 1980 and 2010. Most of these studies were published between 2000 and 2010 and only eight of the 32 studies met the methodological standards for health economic studies [39]. Of the 32 studies, only one pertained to imaging diagnosis [40].

In our literature search, we found a very small number of economic evaluation studies on the subject of MSK ultrasound. One meta-analysis examined the role of MRI for the assessment of soft-tissue and articular disorders of the shoulder and elbow compared to ultrasound, MR arthrography, and CT arthrography [41]. The authors found ultrasound to be the most cost-effective imaging modality for the detection of full-thickness rotator cuff tears with comparable accuracy to MRI. Both MRI and ultrasound were found reliable diagnostic methods for chronic lateral epicondylosis and partial and complete biceps tendon tears and bicipitoradial bursitis. Another retrospective study found that MRI is not cost-effective to investigate nonspecific hip pain in patients aged between 40- and 80-year old [42], whereas in a randomized clinical trial, Sibbitt et al. [43] found that ultrasound-guided intra-articular injections improved clinical outcomes and cost-effectiveness for inflammatory arthritis as compared to intra-articular injections based on anatomic landmarks.

## Conclusions

Besides the importance of practice standards, certification, and accreditation to ensure that MSK ultrasound is ethically and adequately performed in the best interest of patient care, health technology assessment in the form of cost-effectiveness, and cost-utility analysis using model or trial-based data is mandatory in the context of healthcare budgets restraints. Use of imaging must be shown to improve patient outcomes and be cost-effective. Current evidence recommends musculoskeletal ultrasound as the primary imaging modality in the investigation of rotator cuff disease because of its high diagnostic accuracy and its cost-effectiveness when compared to MRI, but studies show that physicians do not follow these recommendations. Incentives, computerized tools and policies aiming at ensuring the application of imaging guidelines among physicians are mandatory. Self-referral should also be controlled as this practice has been shown to encourage inappropriate use of imaging.
